# Modulation of SOX2 expression delineates an end-point for paclitaxel-effectiveness in breast cancer stem cells

**DOI:** 10.1038/s41598-017-08971-2

**Published:** 2017-08-23

**Authors:** Pritha Mukherjee, Arnab Gupta, Dhrubajyoti Chattopadhyay, Urmi Chatterji

**Affiliations:** 1 Department of Zoology, University of Calcutta, Kolkata, India; 2 Saroj Gupta Cancer Care and Research Institute, Kolkata, India; 3 Department of Biotechnology, University of Calcutta, Kolkata, India; 4 Present Address: Amity University Kolkata, New Town, India; 5 Centre for Research in Nanoscience and Nanotechnology, University of Calcutta, Kolkata, India

## Abstract

Tumor relapse in triple negative breast cancer patients has been implicated to chemoresistant cancer stem cells (CSCs), which under favorable conditions culminate in tumor re-formation and metastasis. Hence, eradication of CSCs during systemic chemotherapy is imperative. CSCs were sorted using immuno-phenotyping and aldefluor assay. Gene expression profiling of normal breast stem cells and breast CSCs from chemo-treated patients were carried out. Silencing SOX2 was achieved by siRNA method. Mammosphere culture and wound healing assays were carried out to assess efficacy of CSCs. Microarray analysis revealed elevated expression of SOX2, ABCG2 and TWIST1, unraveling an intertwined pluripotency-chemoresistance-EMT axis. Although paclitaxel treatment led to temporary arrest of cell migration, invasiveness resumed after drug removal. The ‘twist in the tale’ was a consistently elevated expression of TWIST1, substantiating that TWIST1 can also promote stemness and chemoresistance in tumors; hence, its eradication was imperative. Silencing SOX2 increased chemo-sensitivity and diminished sphere formation, and led to TWIST1 down regulation. This study eventually established that SOX2 silencing of CSCs along with paclitaxel treatment reduced SOX2-ABCG2-TWIST1 expression, disrupted sphere forming capacity and also reduced invasiveness by retaining epithelial-like properties of the cells, thereby suggesting a more comprehensive therapy for TNBC patients in future.

## Introduction

On a global scale, breast cancer is the most frequently diagnosed cancer, accounting for 29% of total cancer cases, and the leading cause of cancer deaths amongst females^1^. Data suggests that 1 in 28 women in urban India and 1 in 64 women in rural India are at a risk of developing breast cancer^2^. Despite advances in early detection, approximately 30% of all patients often turn up with recurrence of the disease within 2 to 5 years after completion of treatment^3^. To offer treatment with increased efficacy and low toxicity, selective therapies based on molecular characteristics of the tumor is therefore necessary to prevent disease relapse^3, 4^.

Amongst the different types of tumors of the breast, triple negative breast cancers (TNBC) evolved to be of prominent occurrence, especially in patients from India and Bangladesh, and now reported to be amongst the top contenders of breast cancer cases in the US^1, 5, 6^. The major caveat in pathologic complete response of TNBC is their relatively poor prognosis and high rates of local, regional or distant recurrences^7, 8^. Tumor relapse may be implicated to the meager population of cancer stem cells (CSCs), which contribute to relatively low survival rates in these patients^9^. CSCs constitute self-sustaining cells which under conducive conditions lead to development of heterogeneous lineages, and eventually culminate in tumor re-formation and metastasis^10, 11^. CSCs share many properties of normal stem cells (NSCs) including a long lifespan, relative quiescence, and resistance to drugs through the expression of drug efflux pumps, an active DNA-repair capacity and resilience to apoptosis. Such a population of drug-resistant pluripotent cells can therefore survive chemotherapy and re-populate the tumor^12^.

The persistence of CSCs through chemotherapy renders them invincible components of tumors. A strong relationship exists between pluripotency and chemoresistance, tethered to epithelial-to-mesenchymal transition (EMT)^13, 14^ which ultimately governs the aggressive nature of TNBCs. High levels of ATP-binding cassette (ABC)-transporters in CSCs render them resistant to various chemotherapeutic agents^15, 16^ and can explain resistance and tumor recurrence to traditional anti-cancer drugs. Hence, selective inhibition and/or eradication of breast cancer stem cells (brCSCs) during systemic chemotherapy would provide TNBC patients a more complete therapeutic option. Our aim, therefore, was to define mechanisms that would render the brCSCs more receptive to the effects of conventional chemotherapeutic drugs, like paclitaxel (Pax). Since genes other than ABC-transporters may participate in development of chemoresistance in CSCs^17, 18^ identifying additional factors that aid ABC-transporters in conferring chemoresistance also need to be identified. In the current study, we have shown that silencing SOX2 along with administration of Pax can render the brCSC population less aggressive, with regard to chemo-resistance and migration, via modulation of ABCG2 and TWIST1 expression.

## Results

### Chemotherapy enriches brCSCs in human triple negative breast tumors

**Figure 1:**
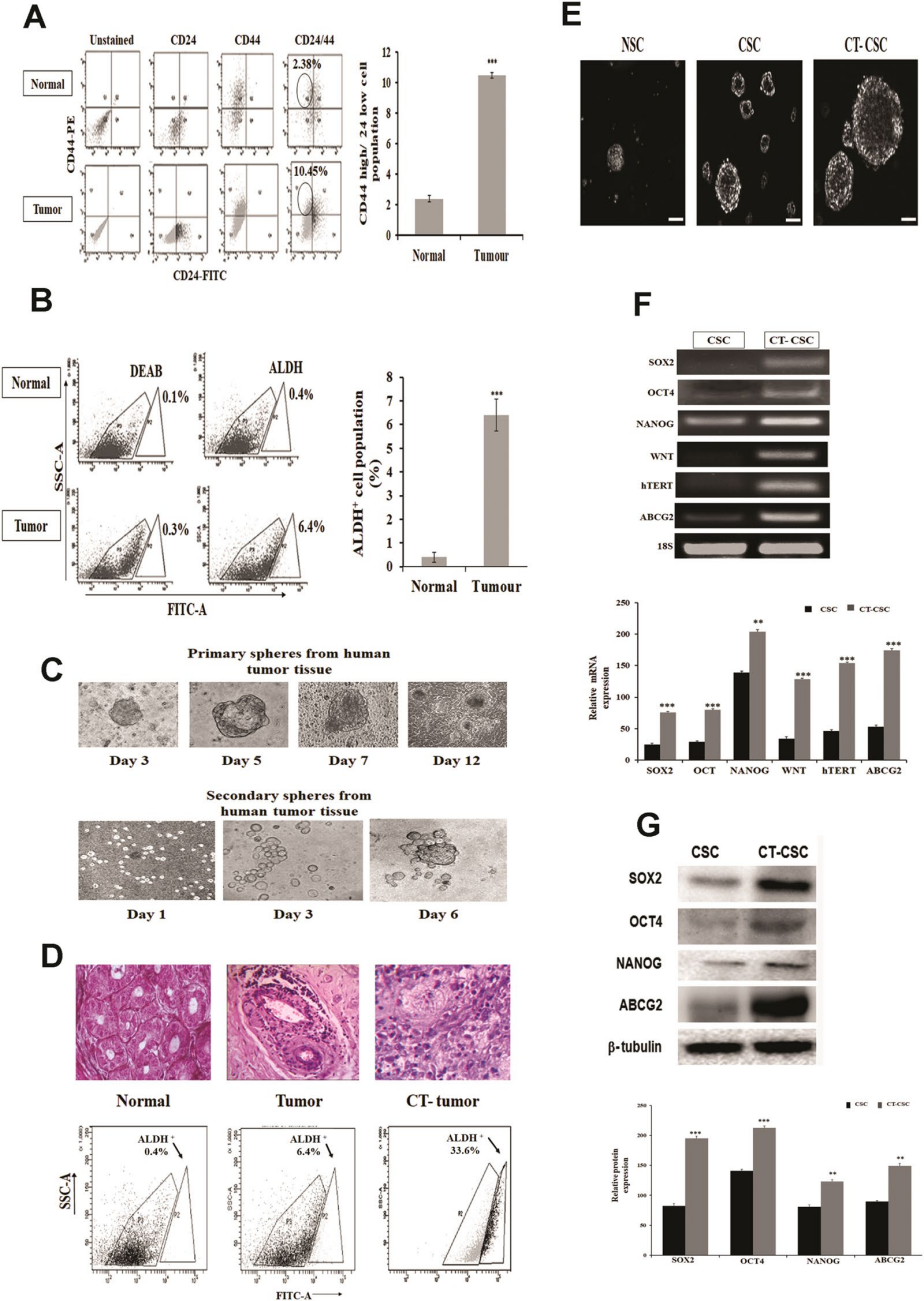
Breast cancer stem cells show higher expression of pluripotency genes and chemoresistance marker. (**A**) Immunesorting of normal mammary tissues and breast tumor tissues showing 10.45% cells with CD44^+^/CD24^−^ phenotype in tumors as compared to 2.38% in normal tissues (n = 50). (**B**) Aldefluor assays from patient tumors show a higher percentage of ALDH^+^ cells (6.4%) as compared to normal tissues (0.4%) (n = 50). (**C**) Representative images showing the morphology of primary and secondary mammospheres grown in serum-free cultures from TNBC tumors (20X magnification; n = 30). (**D**) Representative hematoxylin-eosin staining of normal mammary tissue and TNBC tumor obtained after MRM surgery from naïve (Tumor) and chemo-treated (CT-Tumor) patients. Aldefluor assays show 33.6% ALDH^+^ cells in the CT-tumor (n = 30). (**E**) Mammospheres formed from sorted normal stem cells (NSC), cancer stem cells from untreated TNBC tumor (CSC) and cancer stem cells from TNBC CT-tumor (CT-CSC) after 7 days of culture (20X magnification). (**F**) Expressions of *SOX2, OCT4, NANOG, WNT, hTERT* and *ABCG2* in sorted ALDH^+^ populations of TNBC tumor (CSC) and chemo-treated TNBC tumor (CT-CSC). 18S was used as the endogenous control. The color bars represent expression of markers in CSCs (darker bars) versus chemo-treated CSCs (lighter bars) (**G**) Western blot analyses of SOX2, OCT4, NANOG and ABCG2 in breast cancer stem cells isolated from TNBC tumor (CSC) and chemo-treated TNBC tumor (CT-CSC). The color bars represent expression of markers in CSCs (darker bars) versus chemo-treated CSCs (lighter bars). Data are expressed as mean ± SEM of three independent experiments. Student’s t-test was used to calculate statistical significance. *p < 0.05, **p < 0.01 and ***p < 0.001.

**Figure 2:**
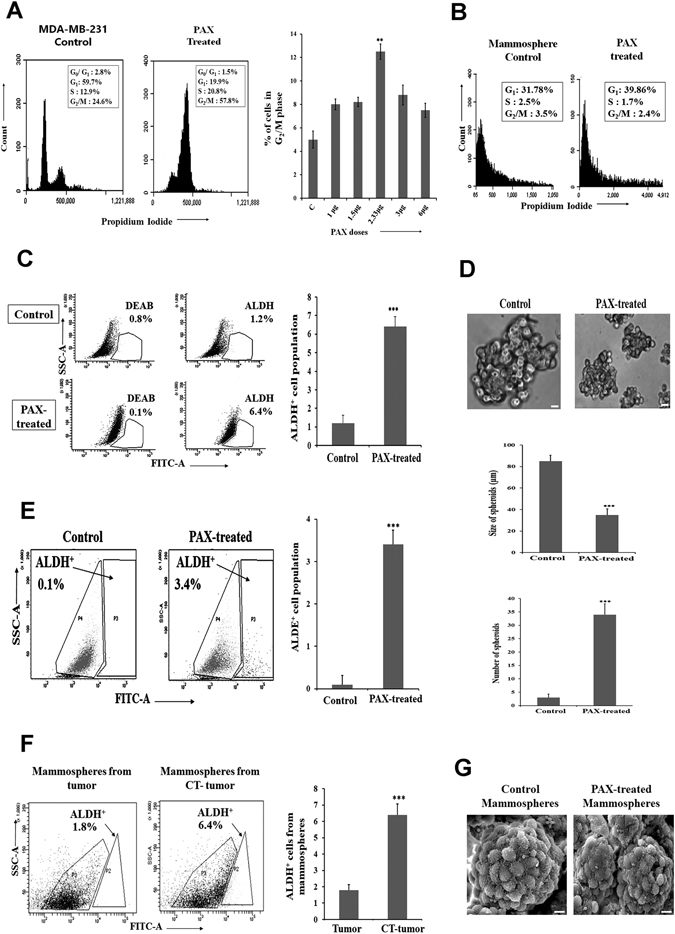
Paclitaxel (Pax) treatment enriches cancer stem cells in MDA-MB-231 cells. (**A**) Cell cycle analyses by flow cytometry of MDA-MB-231 cells showed a G_2_/M phase arrest after 48 hours of Pax treatment (2 nM). Histogram showing the population of MDA-MB-231 cells in G_2_/M phase after treatment with different doses of Pax (1–6 nM). (**B**) Cell cycle analysis of mammospheres from MDA-MB-231 before and after treatment with Pax. (**C**) Aldefluor analysis of MDA-MB-231 before and after treatment with Pax. (**D**) Pax-treated mammospheres from MDA-MB-231 indicated increase in number and decrease in size of the spheres. Scale bar 10 µM. (**E**) Pax treatment increases the ALDH^+^ cells in MDA-MB-231 mammospheres (3.3%). (**F**) Increase in the ALDH^+^ cells (6.4%) were also seen in mammospheres from TNBC CT-tumor (chemo-treated) as compared to an untreated tumor (1.8%). (**G**) Scanning electron microscopy images of mammospheres from control and Pax-treated MDA-MB-231 under 2 µm magnification. Data are expressed as mean ± SEM of three independent experiments. Student’s t-test was used to calculate statistical significance. *p < 0.05, **p < 0.01 and ***p < 0.001.

Both immune-sorting and aldefluor assays revealed that human breast tumors harboured a higher population of both CD44^+^/CD24^−^ (Fig. 1A) and ALDH^+^ (aldehyde dehydrogenase^high)^ cells (p < 0.001), compared to normal tissues (Fig. 1B). Chemo-treated patient tumors (CT-Tumor) showed a higher percentage of ALDH^+^ cells (73.2%) as compared to untreated naïve tumors (14.7%; Supplementary Fig. 1). Immunophenotyping of CD44^+^/CD24^−^ populations in naïve tumors and chemo-treated tumors from patients undergoing MRM in comparison to the normal mammary tissue showed a differential count of this subset in the cancer stem cell population with chemo-treatment augmenting their numbers (Supplemenatry Fig. 2). Sphere forming assays with brCSCs from human tumors confirmed their self-renewal property. Efficacy of CSCs was further ascertained when primary mammospheres generated secondary spheres within 6 days of re-seeding the Day 7 primary spheres (Fig. 1C). Interestingly, ALDH^+^ cells elevated enormously (33.6%; p < 0.001) in tumors from TNBC patients who had undergone pre-surgery chemotherapy, compared to the untreated patients (6.4%; p < 0.01) (Fig. 1D). The sphere forming efficiency was more pronounced in case of CT-CSCs (chemo-treated CSCs) compared to CSCs from untreated patient tumors (Supplementary Fig. 3). In addition, the size of mammospheres was larger when derived from chemo-treated tumors in comparison to the non-chemo treated naïve tumors (Fig. 1E). ALDH^+^ cells (indicating putative brCSCs) from chemo-treated tumors expressed higher levels of stem cell markers compared to ALDH^+^ cells from untreated tumors, both at the transcriptional (Fig. 1F) and translational levels (Fig. 1G). Expressions of *SOX2, OCT4, WNT, NANOG, ABCG2 and ALDH1A1* was assessed both at transcriptional and translational levels from spheroids and sorted populations of CD44^+^/24^−^ and ALDH^+^ cells of CT-TNBC tumor (CSC) versus the whole tumor (Supplementary Fig. 1).

### Paclitaxel treatment augments brCSCs in MDA-MB-231 cells and mammospheres

To confirm the above findings *in vitro*, we simulated chemo-treatment of human TNBC tissues by treating the MDA-MB-231 triple negative breast cancer cells and mammospheres formed from MDA-MB-231 cells with Pax. The optimum dose of Pax was determined by flow cytometry after exposing the cells to different doses of Pax (1 nM to 6 nM) for 48 hours. We observed that 2 nM Pax led to significant (p < 0.01) cell cycle arrest of the monolayer cells at the G_2_/M phase (Fig. 2A). However, Pax did not alter the cell cycle status of mammospheres, since cells of the mammospheres which were mostly distributed in the G_0_/G_1_ phase, continued to do so even after drug treatment (Fig. 2B). Aldefluor assay of monolayer cells treated with Pax indicated an increase (>5-fold; p < 0.001) in ALDH^+^ cells (Fig. 2C), compared to the untreated cells. Pax treatment of MDA-MB-231 mammospheres led to a decrease in size and increase in the number of spheres (Fig. 2D), compared to untreated spheres. To assess whether the spheres are essentially formed of CSCs, aldefluor assay of mammospheres prior to and after Pax treatment was carried out. The results showed a 3-fold increase in ALDH^+^ cells in spheres treated with Pax (p < 0.001) (Fig. 2E). This observation conformed to the results of aldefluor assay of mammospheres from untreated and chemo-treated human tissues, where chemo-treatment led to an increase (3.5-fold; p < 0.001) in the number of ALDH^+^ cells (Fig. 2F). CD44^+^/CD24^−^ analysis from MDA-MB-231 spheres with PAX treatment also established the contribution of paclitaxel in increasing the CD44^+^/24^−^ population (Supplementary Fig. 2).

SEM analysis of Pax-treated and untreated spheres indicated that untreated spheres show compact rosette arrangement of cells adhered to each other, whereas after drug treatment, the size of the spheres were reduced, and the structural integrity and compactness was markedly compromised during sphere formation (Fig. 2G).

### Differential gene expression in brCSCs isolated from normal breast tissues and chemotreated triple negative breast tumors

**Figure 3:**
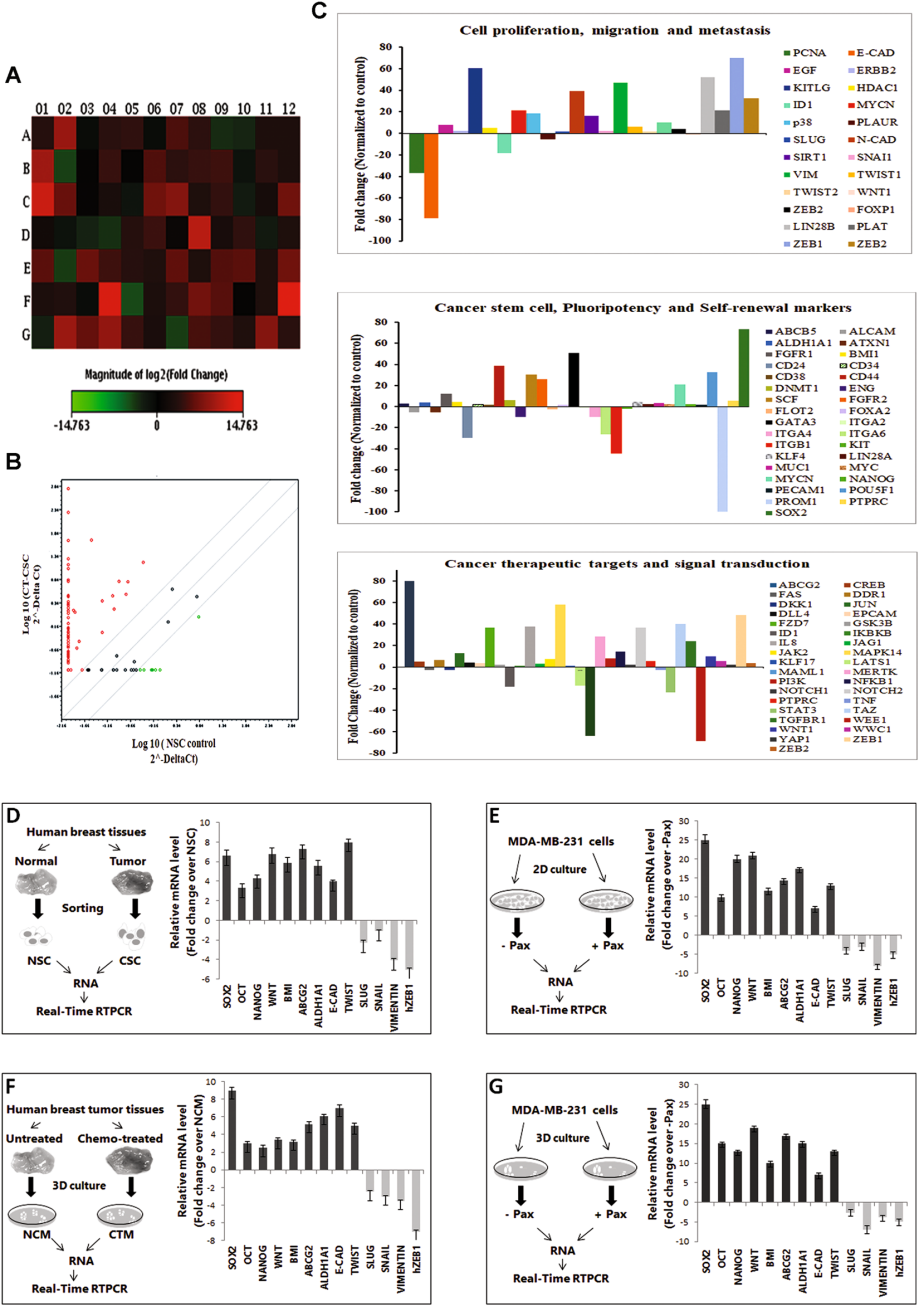
Microarray analysis elucidates differentially expressed stem cell-related genes in normal breast stem cells (NSC) and breast cancer stem cells from chemo-treated TNBC patients (CT-CSC). (**A**) Heat Map of the 96 genes in the RT^2^ profiler array plate for Cancer Stem Cells showing the fold regulation (log2 fold change) of various genes from the chemo-treated CSCs of TNBC tumors normalized to normal stem cells (NSCs) from the same patient. (**B**) A scatter plot showing the normalized expression of all genes on the array between CT-CSC and NSC to visualize large gene expression changes. The central line indicates unchanged gene expression. (**C**) Multigroup plots to represent the expression of selected genes from the array with grouping under three different functional categories. (**D**) qRT-PCR analysis for stemness, chemoresistance and EMT genes from ALDH + population of normal (NSC) and chemo-treated TNBC tumor (CSC). (**E**) Expression of genes from Pax-treated (+PAX, 2 nM) MDA-MB-231 compared to control cells (−Pax). (**F**) Expression of genes in spheroids cultured from chemo-treated TNBC tumor (CTM) compared to spheroids from untreated tumor (NCM). (**G**) Differential expression of genes from Pax treated (+Pax, 2 nM) mammospheres of MDA-MB-231 cells compared to untreated (−Pax) mammospheres. The error bars represent standard error of the mean of 20 individual tumors from human patients (n = 20). Data are expressed as mean ± SEM of three independent experiments. Student’s t-test was used to calculate statistical significance. *p < 0.05, **p < 0.01 and ***p < 0.001.

To identify specific genes responsible for enhanced stemness of brCSCs harbored within tumors in patients, a DNA microarray was carried out with NSCs and brCSCs from chemo-treated TNBC patients. The array determined the levels of differentially expressed genes belonging to various functional cohorts. According to data mining from the bioinformatics databases, these genes were linked to CSC proliferation, self renewal, pluripotency, asymmetric cell division, migration and metastasis, which helped facilitate both CSC characterization, as well as, identify targets of therapeutics currently being tested. A set of controls present on this array enabled data analysis using the ∆∆CT method of relative quantification. Using real time PCR we analyzed the expression of a focused panel of genes related to stem cells by Log_10_ RQ using SYBR^®^ green relative quantification assay. DNA microarray analysis demonstrated 84 different genes with log ratios of >2. The positive controls, as well the housekeeping genes, were expressed in all samples, while the negative controls were not. The heat map of genes with log ratio >2 (Fig. 3A), a scatter plot representing normalized gene expression (Fig. 3B), and multigroup plots representing various functional cohorts (Fig. 3C) have been shown.

To confirm the microarray data, differential expression of pluripotency and chemoresistance genes were performed in the following samples: (i) Breast NSCs and brCSCs from chemo-treated human tumors, (ii) Pax treated and untreated MDA-MB-231 cells, (iii) mammospheres from non-chemo (NCM) and chemo-treated human tissues (CTM) and (iv) mammospheres from MDA-MB-231 cells treated without and with Pax. Higher expressions of *SOX2, OCT4, NANOG, WNT, BMI1, ABCG2, ALDH1A1, E-CADHERIN* and *TWIST1* and lower expressions of *SLUG, SNAIL1, VIMENTIN* and *hZEB1* were consistently observed in the brCSCs as compared to NSCs from sorted human tissues (Fig. 3D) and MDA-MB-231 monolayer cells treated with Pax (Fig. 3E). Similar results were observed in mammospheres that were formed from chemo-treated human tissues (Fig. 3F) and Pax-treated mammospheres from MDA-MB-231 cells (Fig. 3G).

### SOX2 reiterates stemness in triple negative breast cancer

**Figure 4:**
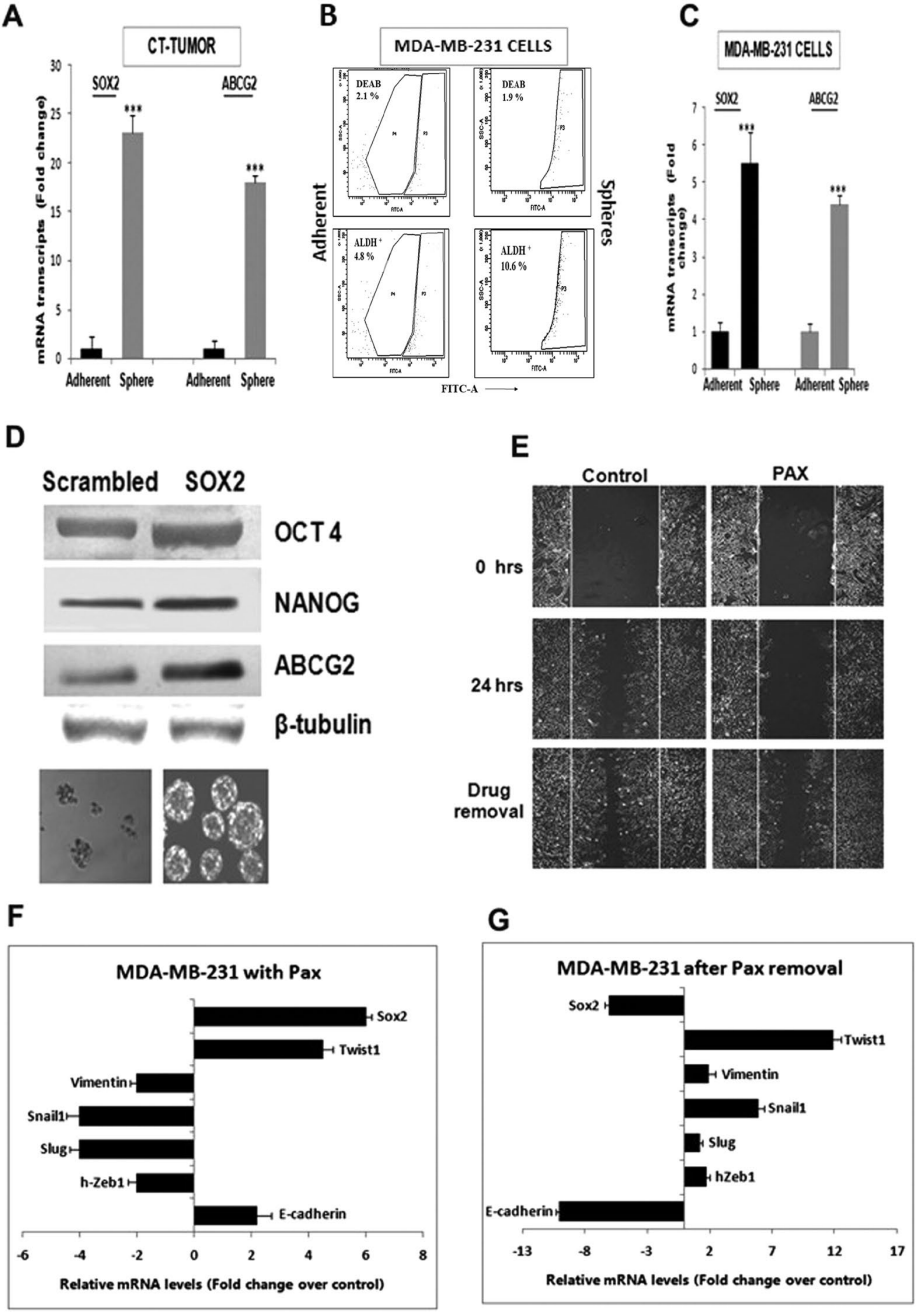
SOX2 is correlated to chemoresistance gene ABCG2 and EMT marker TWIST. (**A**) Higher expression of SOX2 and ABCG2 mRNAs in mammospheres from triple negative breast tumors compared to primary adherent cells from the same chemo-treated (CT) tumor (p < 0.001). (**B**) MDA-MB-231 cells showed a higher ALDH^+^ population in mammospheres compared to adherent cells. (**C**) Higher expression of both SOX2 and ABCG2 was observed in mammospheres developed from MDA-MB-231 cells (p < 0.001). (**D**) Over-expression of SOX2 in MDA-MB-231 cells led to increased expression of OCT4, NANOG and ABCG2 protein, along with robust formation of spheres. β-tubulin was used as the loading control. (**E**) Wound healing assays in MDA-MB-231 cells for 24 hours of Pax treatment (2 nM). Migration of treated cells compared to control after 24 hours of drug removal is shown in the bottom panel. (**F**) Migratory properties indicated by mRNA expression show up regulation of *SOX2, OCT4, WNT, ABCG2, TWIST1, E-CADHERIN* and down regulation of mesenchymal markers *VIMENTIN, SNAIL, SLUG1, hZEB1* after Pax treatment of MDA-MB-231 cells. (**G**) Drug removal showed a decrease in the mRNA expression of all pluripotency genes along with *E-CADHERIN* and a simultaneous increase in the mesenchymal markers. *TWIST1* expression remained high under both conditions.

Amongst the genes related to pluripotency and stemness, the expression of SOX2 was of significant prominence, especially in mammospheres from drug-enriched human triple negative breast tumors and cell lines, as evident in Figs 3F and G. In addition, increased SOX2 expression consistently correlated with an enhanced expression of the chemo-resistance marker, ABCG2, and the EMT marker, TWIST1 (n = 20; p < 0.001), although the expression of the other EMT markers remained significantly low. To reconfirm that increased SOX2 correlated with elevated chemoresistance of the CSC population, chemo-treated (CT) tumors were digested and subjected either to adherent or sphere culture. We observed that expressions of both SOX2 and ABCG2 were higher in mammospheres as compared to the adherent cells (Fig. 4A), indicating enhanced expression of the respective genes in the CSC compartment of human tumor tissues. Concomitantly, MDA-MB-231 cells grown as adherent cells versus spheres showed not only a higher percentage (4.8% versus 10.6%, respectively) of ALDH^+^ cells (Fig. 4B), but also increased expressions of both *SOX2* and *ABCG2* in the spheres (Fig. 4C). To further authenticate that increased SOX2 correlated with increased ABCG2, SOX2 was over expressed in MDA-MB-231 cells. The results indicated concomitant increase in OCT4, NANOG and ABCG2, together with formation of robust mammospheres within 4 days of seeding (Fig. 4D). Next, to substantiate the association of SOX2 with expression of TWIST1 and the other mesenchymal markers, wound healing assays were carried out with MDA-MB-231 cells in the absence (control) and presence (Pax) of drug treatment. Our results indicated that Pax treatment retarded cell migration, thereby reducing wound healing in triple negative breast cancer cells, compared to untreated cells (Fig. 4E). Concomitantly, the expression of SOX2 and EMT markers in cells undergoing migration versus those that were retarded by Pax after 24 hours of instilling the wound revealed that *SOX2* was consistently over expressed in cells treated with Pax (as also observed in Fig. 3E). As expected, expression of the EMT markers viz., *h-ZEB1, SLUG, SNAIL1 and VIMENTIN* were reduced with an increased expression of *E-CADHERIN* (Fig. 4F). Interestingly, the expression of TWIST1 remained high. On removal of the drug, simulating a post-chemotherapy condition, and incubation for another 24 hours, we observed recovery of invasiveness of the Pax-retarded cells, along with increased expression of *hZEB-1*, *SLUG*, *SNAIL1* and *VIMENTIN* and reduced expression of *E-CADHERIN* and *SOX2*. There was, however, no change in expression of *TWIST1*, which remained significantly high (Fig. 4G).

### Silencing SOX2 reduces chemoresistance of breast cancer stem cells ***in vitro***

**Figure 5:**
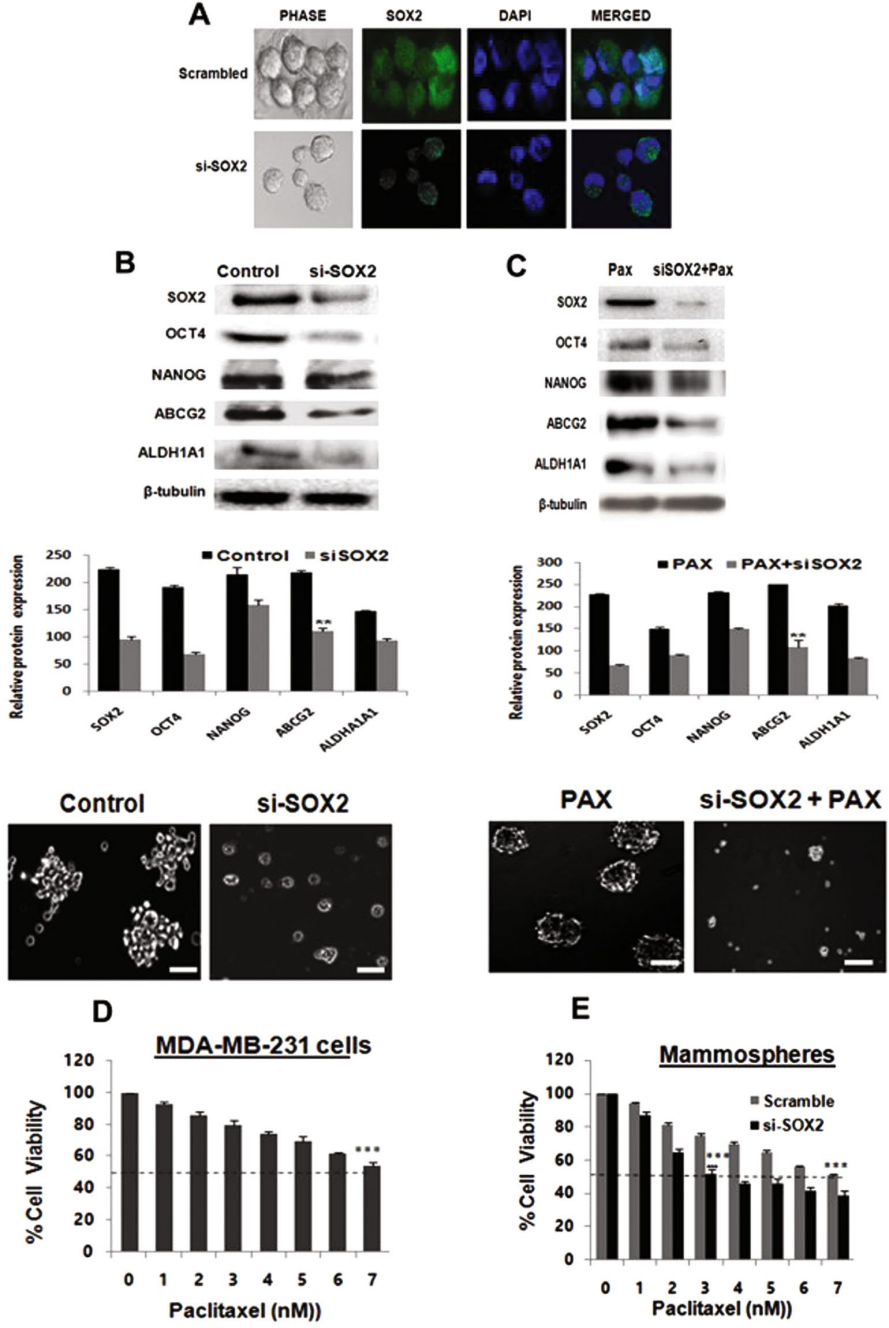
SOX2 silencing decreases chemoresistance and increases paclitaxel sensitivity in breast CSCs. (**A**) Immunofluorescence of SOX2 in mammospheres developed from CT-TNBC treated with siRNA-SOX2 as compared to scrambled controls. (**B**) Silencing SOX2 in CT-TNBC mammospheres showed decreased protein expressions of SOX2, OCT4, NANOG, ALDH1A1, and ABCG2. Si-SOX2-mammospheres also showed deceased sphere forming capacity as compared to scrambled control (Lower panel, 20X magnification). (**C**) Silencing SOX2 in Pax-treated (2 nM) MDA-MB-231 mammospheres results in decreased protein expression of OCT4, NANOG, ALDH1A1. SOX2 down regulation also showed degradation of Pax-enriched mammospheres. (**D**) Cell viability assay of MDA-MB-231 cells after Pax treatment showed 7 nM as the IC_50_ dose. (**E**) Chemosensitivity assay of mammospheres silenced for SOX2 showed increased chemosensitivity to Pax compared to scrambled control. Student’s t-test was used to calculate statistical significance. *p < 0.05, **p < 0.01 and ***p < 0.001.

Since SOX2 over expression consistently correlated with higher expression of ABCG2, we additionally ratified a direct relationship between pluripotency and drug resistance. Consequently, SOX2 was silenced in mammospheres, formed from human tumor tissues (Fig. 5A) which resulted in down regulation of OCT4, NANOG, ALDH1A1, and most importantly, ABCG2 expression (Fig. 5B; p < 0.01), confirming a direct correlation of SOX2 with ABCG2. Silencing SOX2 in TNBC cells impaired their ability of generating spheroids, indicating loss of self-renewal capacity in the *in vitro* system (Supplementary Fig. 4). SOX2 silencing in mammospheres also significantly reduced formation of spheres, indicating diminished self-renewal capacity of the brCSCs (Fig. 5B). Combined effects of silencing SOX2 and Pax treatment on mammospheres indicated that contrary to Pax treatment of unsilenced mammospheres, SOX2-silenced mammospheres from human tumor tissues treated with Pax showed significantly reduced expressions of ABCG2, OCT4, NANOG and ALDH1A1, concomitant with degradation of silenced mammospheres (Fig. 5C, p < 0.01).

That SOX2 plays an important role in chemo-resistance of cancer stem cells was further demonstrated by chemo-sensitivity assays of control and silenced spheres, which were exposed to different concentrations of Pax. MTT assays revealed that the IC_50_ of Pax in MDA-MB-231 cells was 7 nM (Fig. 5D), which was equivalent to mammospheres transfected with scrambled siRNA (Fig. 5E). However, on transfection of the mammospheres with SOX2 siRNA, the IC_50_ reduced to 3 nM, indicating that reduced SOX2 expression could increase chemosensitivity of the stem cell compartment of cancer cells (Fig. 5E).

### Pax treatment confers TWIST1-independent reduction of invasiveness in SOX2-silenced triple negative breast cancer cells

**Figure 6:**
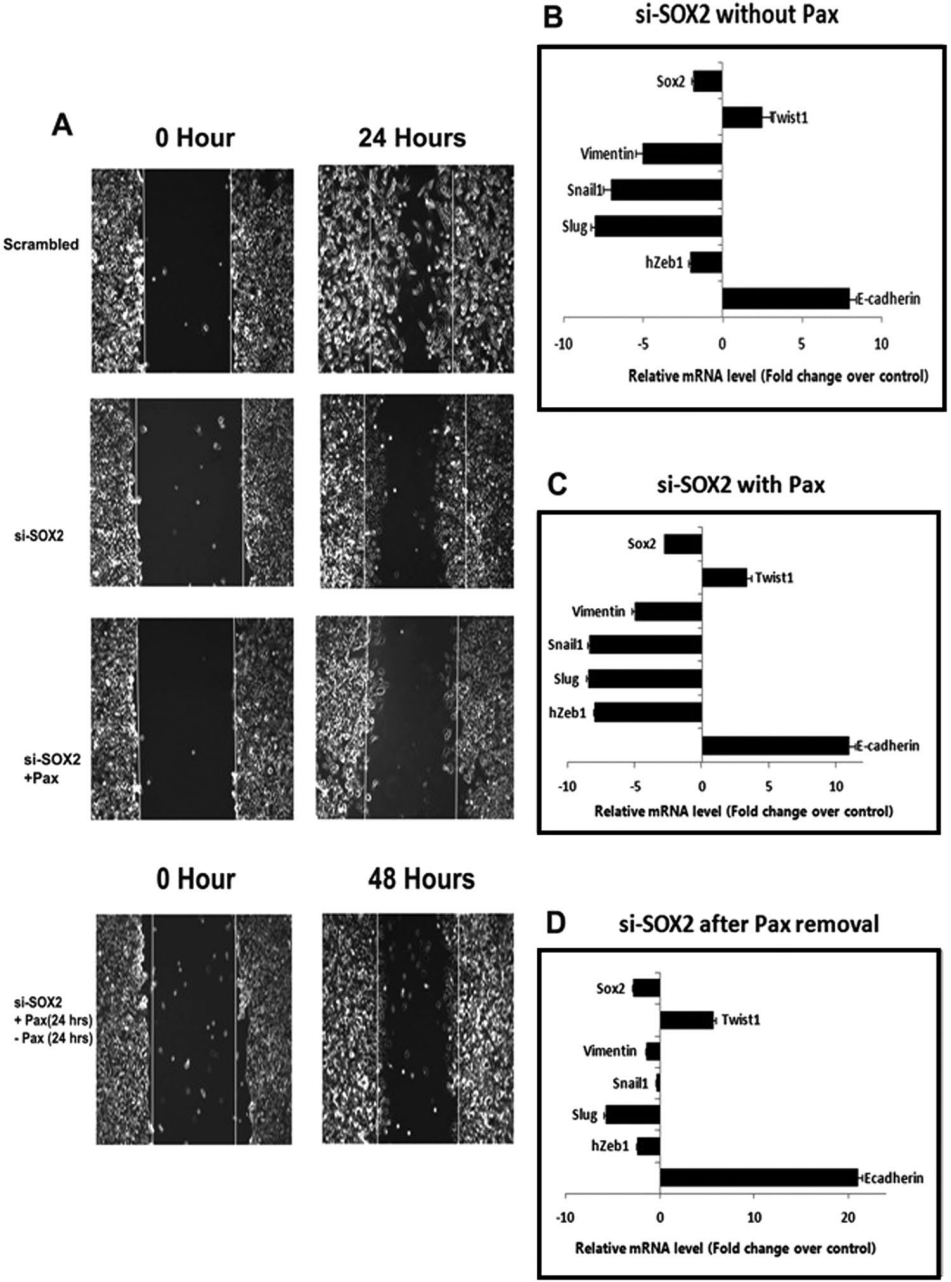
SOX2 silencing inhibits migration in TNBC cells even after Pax withdrawal. (**A**) Wound healing assays showing reduced migration of si-SOX2-treated MDA-MB-231 cells compared to unsilenced cells after 24 hours. Inhibition of migration continued 24 hours after drug removal (2 nM PaX) in SOX2 silenced MDA-MB-231 cells. (**B–D**) qRT-PCR analyses of pluripotency, chemoresistance and EMT genes from each of the above sets. Data are expressed as mean ± SEM of three independent experiments. Student’s t-test was used to calculate statistical significance. *p < 0.05, **p < 0.01 and ***p < 0.001.

Although Pax treatment temporarily arrested MDA-MB-231 cell migration independent of TWIST1 expression, invasion was seen to resume after drug removal (Fig. 4G). In this context, we subsequently verified whether SOX2 silencing would have more lasting migration arrest effects on MDA-MB-231 cells under similar conditions. Wound healing assays indicated that compared to the unsilenced cells, SOX2 silencing significantly prevented migration of MDA-MB-231 in adherent cultures (Fig. 6A; p < 0.01). Reduced invasiveness was more prominent when SOX2-silenced cells were treated with Pax. Interestingly, migration of SOX2-silenced cells continued to remain arrested even after 24 hours of Pax removal (Fig. 6A) by almost 85%, unlike Pax removal from cells that were not silenced for SOX2 (Fig. 4G). To confer the magnitude of migratory arrest we also compared SOX2-silenced MDA-MB-231 with scrambled cells, both treated with Pax for 24 hrs followed by drug removal regimen and assessment of wound healing assay (Supplementary Fig. 5). qRT-PCR indicated concomitant reduced expression of *VIMENTIN, SLUG, SNAIL1* and *h-ZEB1* with a very sharp increase in *E-CADHERIN*. Expression of *TWIST1* however remained significantly high in the SOX2-silenced MDA-MB-231 cells, both during and post-Pax treatment (Fig. 6B–D).

### SOX2 silencing together with Pax treatment reverses TWIST1 expression in breast CSCs

**Figure 7:**
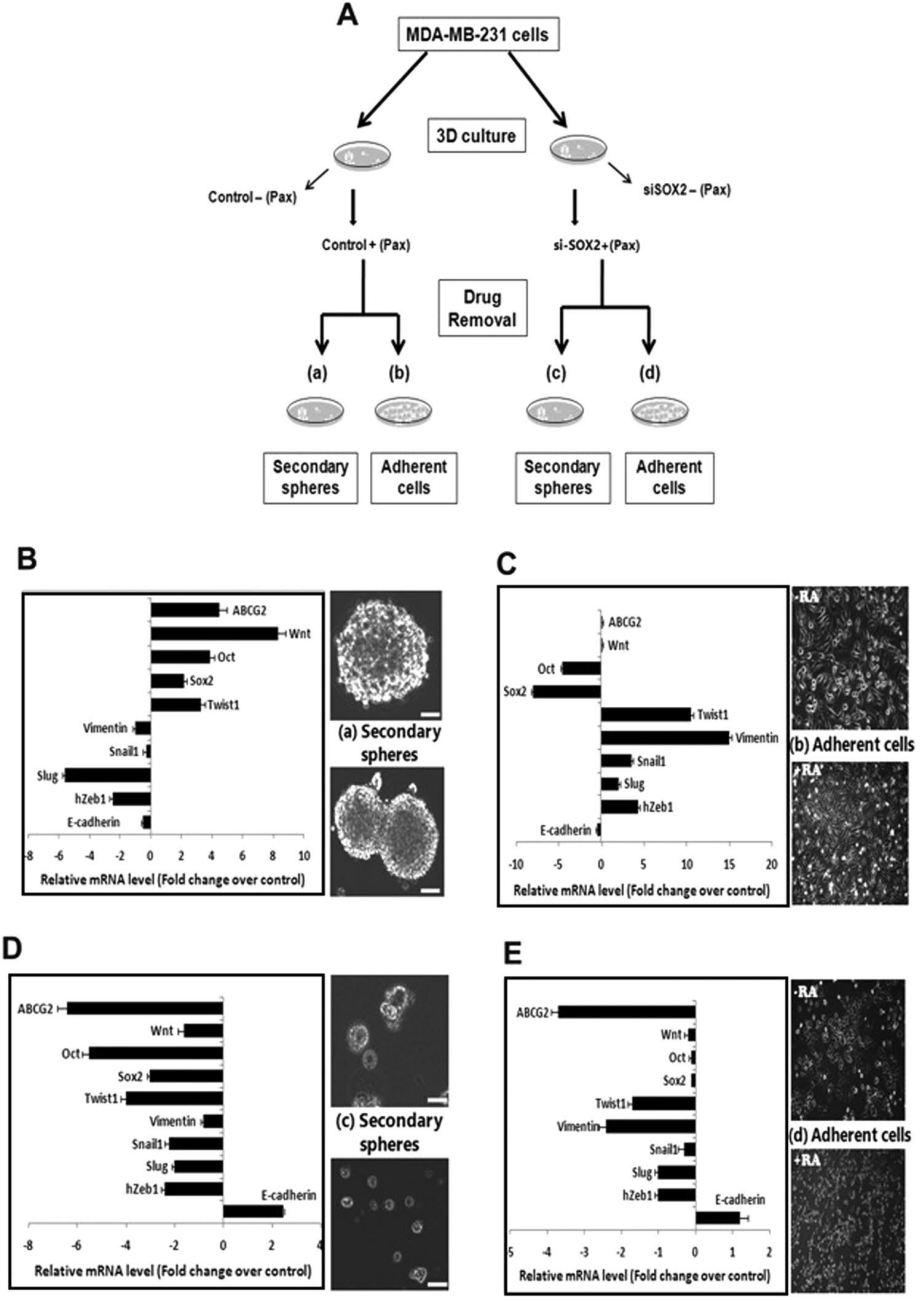
SOX2 silencing and Pax treatment of mammospheres prevent differentiation of CSCs and down regulate TWIST1. (**A**) A diagrammatic representation of the experiment depicting Pax-treatment of silenced spheres which were re-plated to grow as secondary spheres or as adherent cells after drug removal. (**B**) MDA-MB-231 mammospheres were treated with Pax (2 nM) and reseeded as secondary spheres after drug removal. qRT-PCR analysis for pluripotency, chemoresistance and EMT genes are shown. (**C**) Pax-treated MDA-MB-231 mammospheres re-seeded as adherent cultures after drug removal and real-time PCR analysis for the representative genes are shown. (**D**) Pax-treated SOX2-silenced mammospheres reseeded as secondary spheres after drug removal showed decreased expressions of *SOX2, OCT4, WNT, ABCG2, VIMENTIN, SLUG, SNAIL1, hZEB1 and TWIST1*. (**E**) Paclitaxel-treated SOX2-silenced mammospheres reseeded as adherent cultures after drug removal with (+) or without (−) retinoic acid (10 µg/µl). qRT-PCR analysis revealed increased *E-CADHERIN* expression and reduced expression of EMT markers including *TWIST1*. Data are expressed as mean ± SEM of three independent experiments. Student’s t-test was used to calculate statistical significance. *p < 0.05, **p < 0.01 and ***p < 0.001.

The above observations were indicative of an apparent lack of involvement of TWIST1 during migration of both Pax-treated MDA-MB-231 cells and SOX2-silenced MDA-MB-231 cells. However, its expression in the CSC population under similar conditions necessitated clarification. Subsequently, unsilenced and SOX2-silenced spheres were collected after 24 hours of Pax-treatment, washed to remove the drug and replated to grow either as secondary spheres or as adherent cells (Fig. 7A). Pax-treated secondary spheres indicated higher expression of *SOX2, WNT, OCT* and *ABCG2*, and reduced expression of *VIMENTIN, SLUG, SNAIL1* and *h-ZEB1*, indicating retention of self renewal properties. Expression of *TWIST1* remained high in the spheres even after drug removal (Fig. 7B). Spheres that were grown under adherent conditions, in the absence or presence of retinoic acid^19^, differentiated and propagated as a monolayer, irrespective of retinoic acid (Fig. 7C). Expression analysis of these cells indicated elevated *SLUG, SNAIL1*, *h-ZEB1* and *VIMENTIN*, reduced *E-CADHERIN* and a consistently high expression of *TWIST1* (Fig. 7C). However, when SOX2-silenced mammospheres were subjected to similar conditions, the most prominent observation, in addition to disintegrated sphere formation, was down regulation of TWIST1 expression in both secondary spheres and cells cultured under adherent conditions (Fig. 7D and E), along with the other EMT markers. Interestingly, the expression of E-cadherin was restored significantly in both secondary spheres and cells grown in monolayer, together with a more epithelial-like morphology of the cells and limited propagation potential (Fig. 7E).

## Discussion

Despite recent advances in the treatment of triple negative breast tumors, the incidence of distant relapse remains high^20^, necessitating novel therapies to surmount the existing paradigm. Cancer recurrence has chiefly been attributed to the impetuous proliferation of CSCs which are not eliminated by conventional chemotherapy, primarily because of elevated expression of drug efflux pumps^21^. In addition, the fact that chemotherapy enriches the CSC population within a tumor poses a greater threat for the patient^22^. Therefore, modulation of factors responsible for elevating expression of drug transporters points toward a more effective and complete cure for TNBC patients.

Screening a panel of factors known to regulate pluripotency, cell invasiveness/EMT, drug resistance and associated signaling components in NSCs and brCSCs isolated from chemotreated tumors of TNBC patients revealed increased transcript levels of stemness markers, in particular SOX2. Several studies have indicated SOX2 as an important marker for stem and progenitor cells^23, 24^. Recently, SOX2 was found to act as an oncogene in some epithelial cancers^25^, promotes invasiveness of tumor cells in glioma^26^ and is an indicator of poor prognosis in patients with HNSCC^27^. SOX2 has also been linked to drug resistance in several studies^28–31^, though its precise role in triple negative breast tumors has not been elucidated till date. Since SOX2 expression was significantly elevated in the chemo-treated patient samples, we assessed the involvement of SOX2 in increasing chemoresistance and migratory properties of brCSCs. Elevated expression of SOX2 correlated to higher ABCG2 expression in mammospheres from triple negative human tumors compared to primary adherent cells, additionally ratified by adherent (2D) versus sphere (3D) culture of MDA-MB-231 cells. Overexpressing SOX2 in adherent cells and mammospheres confirmed enhanced expression of stemness and drug-efflux markers with accelerated formation of robust mammospheres, affirming self-renewal and chemo-resistance properties of the brCSCs. On the other hand, silencing SOX2 in mammospheres, followed by paclitaxel treatment, to ascertain the effect of combinatorial therapy, could successfully inhibit mammosphere formation and obliterate persistence of stemness properties. Down regulation of SOX2 could additionally help overcome chemo-resistance of brCSCs, as indicated by reduced ABCG2 expression and increased sensitivity to paclitaxel.

The identification of epithelial-mesenchymal plasticity of brCSCs provided another level of complexity regarding development of strategies to eliminate these lethal seeds of breast cancer^32^. In order to resolve the fate of brCSCs within a tumor prior to, during and after chemotherapy, we determined the association between SOX2, cell migration and expression of EMT markers. Wound healing assays revealed that paclitaxel was effective in arresting the migration of triple negative breast cancer cells associated with reduced expression of mesenchymal markers and up regulated *E-CADHERIN* expression. However, simulation of a post-chemotherapeutic condition created by drug removal from the treated cells resulted in restoration of migratory properties similar to control cells within 24 hours of Pax withdrawal. This finding may be implicated to the fact that Pax temporarily prevented cell migration, albeit defying the role of *TWIST1*, expression of which remained high. The fact that drug removal reinstated migration could be possible since the brCSCs which survived Pax treatment differentiated into cancer cells, as evident by down regulation of *SOX2*, and promoted cell migration after Pax withdrawal.

The success of this study unveiled when SOX2-silenced MDA-MB-231 cells were exposed to Pax. Although silencing efficiently retarded migration in the absence of Pax, restricted migration was more pronounced in the presence of Pax, confirming that silencing SOX2 together with Pax treatment was a more effective therapeutic possibility. Interestingly, unlike unsilenced cells where Pax removal restored migratory properties, migration continued to be restricted in SOX2-silenced cells even after drug removal. This further confirmed a more sustained effect of the drug and possible prevention of metastasis even when conducive conditions resumed after chemo-treatment.

What was perplexing in these experiments was the persistently high expression of TWIST1 and its apparent lack of EMT-like properties during migratory arrest of MDA-MB-231 cells, even after paclitaxel treatment of silenced cells. Although TWIST1 has been reported to be one of the master regulators of invasiveness and EMT^33^ and resistant to microtubule-disrupting agents, including paclitaxel^34^, it is interestingly reported to be associated with stem cell maintenance and stemness properties^35^. Chemotherapeutic agents can increase TWIST1 expression in carcinoma cells, and cause drug resistance or decrease sensitivity to drugs like paclitaxel, vincristine, and taxol^36^. There is also evidence that expression of TWIST1 is regulated by SOX2^37^. This also led us to infer that although prominently known to be one of the key promoters of EMT and invasiveness in a number of cancer types, the role of SOX2-dependent TWIST1 in maintaining stemness was more prominent when SOX2 expression was high in brCSCs. However, the precise mechanism underlying the resolution as to whether SOX2 binds to the TWIST1 gene promoter is still under investigation, and it is possible that TWIST1 is differently regulated in the presence and absence of SOX2, when other factors influence its EMT property.

Since SOX2 modulation could bring about an arrest in cell migration of MDA-MB-231 cells and have a sustained effect even after drug removal, we elucidated its role in triple negative brCSCs by determining its effect on Pax-treated mammospheres that were re-seeded either as secondary spheres (3D) or as adherent (2D) cultures. This also further confirmed the impassiveness of TWIST1 as an EMT-promoting factor in triple negative brCSCs and defined its role in the survival of the brCSC population. Our study clearly indicated that SOX2-unsilenced mammospheres which survived Pax treatment possessed self-renewal property and gave rise to robust secondary mammospheres. When cultured as monolayer cells, demonstrated low expression of E-CADHERIN and high expression of mesenchymal markers. This indicated the inherent property of the residual brCSCs to differentiate and migrate under favorable conditions, thereby simulating conditions of tumor recurrence. On the contrary, when SOX2-silenced Pax-treated mammospheres were grown as secondary spheres or as adherent cells, we observed down regulation of TWIST1 for the first time, along with elevated expression of E-CADHERIN, loss of self-renewal properties and gain of anti-migratory properties, as evident from significant down regulation of EMT markers.

Taken together, these data suggests that SOX2 and TWIST1 are major regulators of CSC features in human triple negative breast cancers. Specifically, based on our siRNA knockdown experiments and chemosensitivity assays, the SOX2-ABCG2-TWIST1 axis plays a key role in regulating chemoresistance and tumorigenicity in TNBC stem cells. Therefore, obliterating SOX2 expression specifically in brCSCs before or during chemotherapy is a possible approach to eliminate the brCSC population within a tumor, with a promise to prevent post-chemotherapy recurrences in future.

## Materials and Methods

All methods were performed in accordance with the relevant guidelines and regulations. The experimental protocols were based on methods published in reputed journals and therefore approved by Department of Biotechnology, Government of India, Saroj Gupta Cancer Centre and Research Institute, India and University of Calcutta. All patient samples mentioned in this study were procured after obtaining written consent from them.

### Cell lines and reagents

Human breast cancer cell line, MDA-MB-231, was purchased from National Centre for Cell Sciences, India. Aldefluor reagent and collagenase-hyaluronidase mix were from Stem Cell Technologies (USA); HiPerfect transfection reagent and siRNA kit from Qiagen (USA); bovine insulin, epidermal growth factor, and paclitaxel from Sigma Aldrich (USA); B27 and hydrocortisone were from Life Technologies (USA); TRIzol^®^ and superscript cDNA synthesis kit were purchased from Invitrogen (USA); SYBR green/Rox real time PCR kit was from Kapa Biosystems (USA); phycoerythrin (PE)-conjugated CD44 and fluorescein isothiocyanate (FITC)-conjugated CD24 antibodies were procured from BD Biosciences, USA. All other antibodies were either from Abcam (UK) or from Santa-Cruz Biotechnology (USA).

### Human breast tissue samples

Normal and tumor breast tissues were obtained from Saroj Gupta Cancer Care and Research Institute (SGCC&RI), Thakurpukur, India, as per the Institutional Review Board, in accordance with the Institutional Human Ethical Committee, SGCC&RI. All tissue samples were collected after procuring informed consent from patients, the identity of who were not revealed by the Institute, except for their nationality, age, tumor type and status of treatment administered, that is, whether they were subjected to chemotherapy prior to surgery. The breast tumors were exclusively primary-site cancers that were either naïve or had been subjected to chemotherapy prior to surgery. Normal tissues were collected 6 cm away from or diagonally opposite to the tumor site by means of MRM (modified radical mastectomy) or from reduction mammoplasty cases. A total of 30 triple negative breast cancer patient samples have been described in this study. Our study primarily focused on triple negative breast cancers (TNBC; ER^−^, PR^−^, HER2/neu^−^), based on their prevalence in patients who are treated at SGCC&RI, and confirmed by immunohistochemistry reports of the Pathology Division of SGCC&RI. To simulate similar conditions, subsequent studies were carried out in MDA-MB-231 cells.

### Histology and pathological grading

Histologic type of tumors was determined by pathologist according to WHO classification of breast tumors and graded by modified Bloom-Richardson grading system^38^. Immunohistochemical testing for ER, PR, HER2/neu was applied on all cases. HER2/neu-positive slides were scored based on the intensity and percentage of positive cells on a scale of 0 to 3+ . Cases were reported 0 (negative) if no staining or membrane staining in less than 10% of invasive tumor cells was seen.

### Procuring single cell suspension from human breast tissues

Tissues were collected in DMEM containing antibiotics/antimycotics and dissociated enzymatically using 1 mg/ml collagenase-hyaluronidase mix at 37 °C for 16–18 hrs. Cells were separated by centrifugation, strained through cell strainers and either seeded for mammosphere culture or sorted after viability check^39^.

### *In vitro* mammosphere culture from human tissues

After enzymatic digestion, cells were seeded at 2.5 × 10^4^ cells per well in 6-well ultralow attachment plates in DMEM/F12 with 5 μg/mL bovine insulin, 20 ng/mL recombinant epidermal growth factor, B27 supplement, and antibiotic-antimycotic mix. Mammospheres which formed within 7 days were photographed. For serial passaging, mammospheres were enzymatically dissociated into single cells and re-seeded in low attachment plates^40^. Sphere formation efficiency was calculated by dividing the total number of spheres formed by the total number of live cells seeded multiplied by hundred.

### Culture of mammospheres from human cell line

MDA-MB-231 cells were cultured in DMEM supplemented with 10% fetal bovine serum, 50 U/ml penicillin/streptomycin and 2 mM L-glutamine. For 3D sphere culture, cells grown as adherent cultures were disassociated with trypsin/EDTA, washed with PBS and seeded as described above^41^.

### Detection of ALDH^+^ population by flow cytometry

Aldehyde dehydrogenase (ALDH) enzyme activity in viable cells was determined using a fluorogenic dye based aldefluor assay. Briefly, 1 × 10^6^ cells/ml cells were suspended in aldefluor assay buffer containing ALDH substrate (bodipy-aminoacetaldehyde) and incubated for 45 mins at 37°C. As a reference control, the cells were suspended in buffer containing aldefluor substrate in the presence of diethylaminobenzaldehyde (DEAB), a specific ALDH1 enzyme inhibitor. The brightly fluorescent ALDH1-expressing cells (ALDH1^high^) were detected in the green fluorescence channel (520–540 nm) of FACS Aria III (BD Biosciences) and both ALDH^−^ and ALDH^+^ populations were sorted out^42^.

### Immunophenotyping with CD24 and CD44

MDA-MB-231 cells were resuspended in buffer and incubated in the presence of antibodies against PE-conjugated CD44, FITC-conjugated CD24, and their corresponding isotype controls. The stained cells were processed using flow cytometry (BD FACSAria™ III, BD Biosciences, USA). The results were analyzed using BD FACS Diva v6.1.3/v.7 softwares.

### RNA extraction and real-time RT-PCR

Total RNA was extracted from sorted cells and mammospheres using TRIzol^®^. Reverse transcription was performed using the Superscript III cDNA synthesis kit. Gene expression levels relative to those of 18 S were assessed using qRT-PCR and SYBR-green chemistry. Primer sequences and cycling conditions are shown in Table 1. The reactions were run in triplicates and the generated products were analyzed with the step-one analysis software. The data were evaluated as 2^−ΔΔ^Ct (cycle threshold) values. The results were expressed as normalization ratio of the relative quantities of the target mRNAs to those of the control, and the fold difference to the control was used for the comparison.

**Table 1:** Primers and PCR conditions used for qRT-PCR.

Gene	Forward primer 5′→3′	Reverse primer 5′→3′	PCR conditions
*OCT4*	ATCGAGAACCGAGTGAGA	ACACTCGGACCACATCCTT	52°C; 40 cycles
*SOX2*	GGGAAATGGGAGGGGTGCAAAAGAGG	TTGCGTGAGTGTGGATGGGATTGGTGT	61°C; 40 cycles
*NANOG*	TCCTCCTCTTCCTCTATACTAAC	CCCACAAATCACAGGCATAG	52°C; 40 cycles
*WNT*	GGTTCCATCGAATCCTGCAC	GCCTCGTTGTTGTGAAGGTT	53°C; 40 cycles
*BMI1*	GAAATGAAGAGAAGAAGGGA	CCGATCCAATCTGTTCTGGT	53°C; 40 cycles
*ALDH1A1*	GTTGTCAAACAGCAGAGCCGG	TCTTTCCTCCAACTTGCAGC	56°C; 40 cycles
*ABCG2*	CAGGTGGAGGCAAATCTTCGT	TCCAGACACACCACGGATAAA	53°C; 40 cycles
*hTERT*	CGGAAGAGTGTCTGGAGCA	GGATGAAGCGGAGTCTGGA	61°C; 40 cycles
*VIMENTIN*	TACAGGAAGCTGCTGGAAGG	ACCAGAGGGAGTGAATCCAG	64°C; 40 cycles
*TWIST*	TCTTACGAGGAGCTGCAGAC	CTACCGTTCGACGTCGATA	62°C; 40 cycles
*SNAIL*	GGAAGCCTAACTACAGCGAGCT	ACGGTTACGAGTAGACCCT	65°C; 40 cycles
*SLUG*	CTGGTCAAGAAGCATTTCAACGCC	ATGGGTTACCGGAGAGAGGAGAAA	71°C; 40 cycles
*hZEB1*	CAATGATCAGCCTCAATCTGCA	ACCCTAGTTGGTGGTTACC	67°C; 40 cycles
*E-CADHERIN*	GGCGCCACCTGGAGAGA	TGTCGACCGGTGCAATCTT	64°C; 40 cycles
*18 S*	GTAACCCGTTGAACCCCATT	CCATCCAATCGGTAGTAGCG	53°C; 40 cycles

### Protein extraction and western blot analysis

The sorted CSCs were collected in radioimmunoprecipitation assay buffer containing protease inhibitor cocktail. Proteins separated by SDS-PAGE and transferred onto PVDF membrane were probed with primary antibodies. Blots were subsequently incubated with secondary antibodies, and bands detected using chemiluminescence. Blots were analyzed using the Gel Doc XR type imaging system (BioRad). The intensity of bands was quantified using ImageJ software.

### Wound healing migration assay

MDA-MB 231 cells were seeded in 6-well plates and cultured to 80–90% confluence. Confluent monolayers were scraped to generate scratch wounds and incubated at 37°C for 24 hrs with media containing paclitaxel (2 nM). Images were captured at 0 hour and 24 hours using a ZEISS ProgRes CT3 at 20X magnification from five randomly selected fields in each sample. The wound areas were calculated by NIH ImageJ software and the distance between the opposing edges of the wound was measured in micrometers^43^.

### Flow cytometric analysis of DNA content of breast cancer cell lines and mammospheres

Pax-treated and untreated MDA-MB-231 cells and day 7 spheres were collected and fixed with 70% ethanol for 1 hr at 4°C. Following fixation, cells were permeabilized with 0.1% Triton X-100 containing RNase A (20 µg/ml), washed and resuspended in PBS containing propidium iodide (50 µg/ml) and subjected to flow cytometry using BD Accuri C6 (BD Biosciences, USA). The data was analyzed using the BD Accuri C6 software.

### Microarray analysis

After quantification and qualitative analysis of total RNA, 1 µg of RNA was reverse transcribed using single strand cDNA synthesis kit. Relative expression of genes belonging to human CSCs was determined by quantitative PCR using SYBR green based custom designed human PCR array (PAHS-176Z; Human Cancer Stem Cells RT^2^ profile PCR array). Data were analyzed using ^ΔΔ^Ct method provided by SA Biosciences, USA, with normalization of pluripotency genes expression by geometric mean of five housekeeping genes, viz., Glyceraldehyde-3-phosphate dehydrogenase, beta-2-microglobulin, hypoxanthine phosphor-ribosyltransferase 1, ribosomal protein large P0, beta-actin^44^.

### Scanning electron microscopy

Mammospheres were collected by gentle centrifugation and fixed in 2.5% glutaraldehyde for 2 hrs at 4°C. Fixed spheres were washed with 0.2 M phosphate buffer and subsequently dehydrated through ascending grades of ethanol, placed in chilled acetone for 10 mins, and air dried overnight. After critical point drying, the spheres were coated with platinum in a Sputter Coater (Quarm QCES) and finally viewed by SEM (Zeiss EVO-18-Special Edition, Germany).

### Immunofluorescence staining

Staining of intact spheres was done by fixation onto coated slides with 1:1–20 °C pre-chilled Methanol:Acetone. After permeabilization, slides were incubated with primary antibody overnight at 4°C^45^. Fluorophore conjugated-secondary antibody was added and incubated for 60 mins at room temperature. Slides were subsequently stained with DAPI and mounted using antifade, Images were documented using confocal laser scanning microscope (Olympus) and analyzed using FV-10 ASW 3.0 viewer image browser.

### Sox2 overexpression

Using a retroviral transfection system with the PT67 amphotropic packaging cell line, pBabeSOX2-neo and pBabe control-neo plasmids were stably transfected into MDA-MB-231 cells and spheres derived from human normal and tumor tissues to generate SOX2-overexpressing and control cell clones, respectively.

### Small interfering RNA transfection

RNA interference was performed using HiPerfect Transfection Reagent. Human breast CSCs were grown by plating 2 × 10^3^ cells/well in 6-well low-attachment plates. After 24 hrs, cells were transfected with 30ng siRNA against SOX2 or non-targeting siRNA (negative controls). The cells were harvested after 48 hrs and processed for subsequent experiments.

### Pax - chemosensitivity assay

MDA-MB-231 cells and primary spheroids were grown in 96-well plates. Spheres were silenced for SOX2 expression, following which the cells/spheres were treated with paclitaxel at different concentrations (1 nM to 7 nM). Twenty-four hours later, 20 μL of 3-(4,4-dimethylthiazol-2-yl)-2,5-diphenyltetrazolium bromide (MTT) solution (5 mg/mL in PBS) was added to each well, and the plate was incubated at room temperature for 3 hrs. Absorbance was measured on a SpectraMax 190 device (Molecular Devices) at a wavelength of 570 nm.

### Statistical analyses

SPSS version 16.0 (SPSS, Inc., Chicago, IL, USA) was used to analyze the data. All data are expressed as the mean values or as the percentages of control values ± standard error of the mean depending on the experiments performed. Comparisons between two groups were calculated using Student’s t-test (two-tailed, independent). P < 0.05 was considered to indicate a statistically significant difference. Densitometric analyses of western band were quantified by ImageJ software (imagej.nih.gov/ij) and P value was determined by Student’s t-test in GraphPad software. A value of p < 0.05 was considered statistically significant. Cell population after FACS analyses was represented by bar diagrams.

## Electronic supplementary material

Supplementary Information 1
