# Supplementary material for: Modulation of SOX2 expression delineates an end-point for paclitaxel-effectiveness in breast cancer stem cells
Source: Sci Rep. 2017 Aug 23;7:9170. doi: 10.1038/s41598-017-08971-2 (PMC5569040; doi:10.1038/s41598-017-08971-2)
Supplement: Supplementary file 1 — Supplementary Information 1 [file 41598_2017_8971_MOESM1_ESM.doc]

**Modulation of SOX2 expression delineates an end-point for paclitaxel-effectiveness in breast cancer stem cells**

Pritha Mukherjee^1^, Arnab Gupta^2^, Dhrubajyoti Chattopadhyay^3,4^, Urmi Chatterji^*1,5^

^1^Department of Zoology, University of Calcutta, India

^2^Saroj Gupta Cancer Care and Research Institute, India

^3^Department of Biotechnology, University of Calcutta, India

^4^Presently, Amity University Kolkata, New Town, India

^5^Centre for Research in Nanoscience and Nanotechnology, University of Calcutta, India

^*^Corresponding author

Dr Urmi Chatterji

Cancer Research Lab

Department of Zoology

University of Calcutta

35 Ballygunge Circular Road

Kolkata – 700 019. India.

Tel: +91-93393 76535

Email: urmichatterji@gmail.com


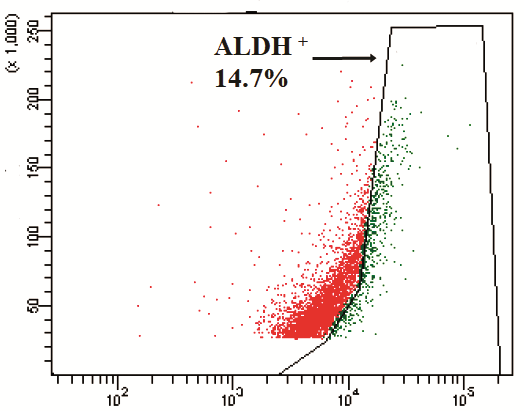

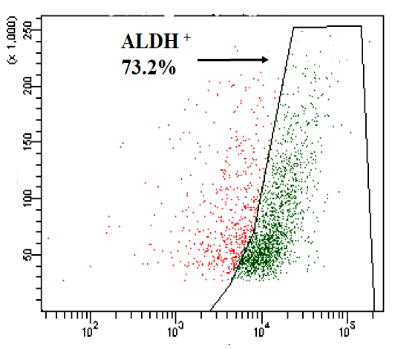


**FITC-A**

**A**

**Tumor**

**CT-Tumor**


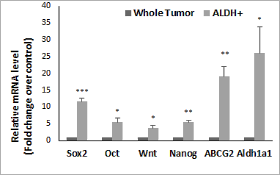

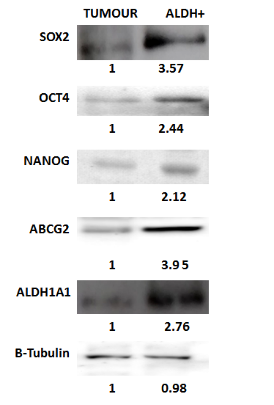

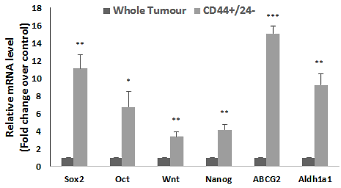

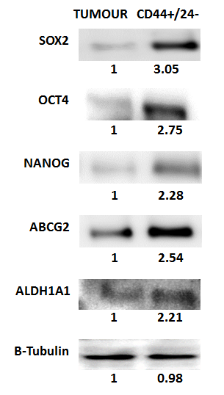

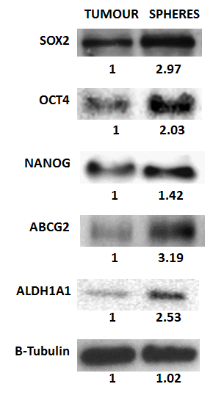


**G**

**F**

**E**

**D**

**C**

**B**


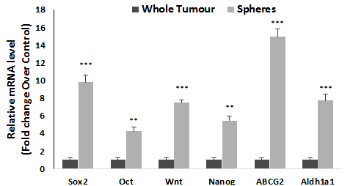


**SSC-A**

**Supplementary Figure 1 (S1): Characterization of stem cell-related markers in spheres, ALDH^+^ or CD44^+^/CD24^-^ versus whole-tumor samples.** (A) Aldefluor assays from chemo-treated patient tumors (CT-Tumor) show a higher percentage of ALDH^+^ cells (73.2%) as compared to untreated naïve tumors (14.7%) (n=30). (B), (D) and (F) show the expressions of *SOX2, OCT4, WNT, NANOG, ABCG2 and ALDH1A1* in sorted populations of CD44^+^/24^-^, ALDH^+^ and spheroids of CT-TNBC tumor (CSC) versus the whole tumor. 18S was used as the endogenous control. The color bars represent expression of markers in whole tumor (darker bars) versus sorted CSCs or spheroids (lighter bars). (C), (E), (G) Western blot analyses of SOX2, OCT4, NANOG, ABCG2 and ALDH1A1 in sorted CSC populations of CD44^+^/24^-^, ALDH^+^ and mammospheres from CT-TNBC tumor versus the whole tumor. Quantitative expression of markers is indicated by numerical values in the figure. Data are expressed as mean ± SEM of three independent experiments. Student’s t-test was used to calculate statistical significance. *p<0.05, **p<0.01 and ***p<0.001.

**A**


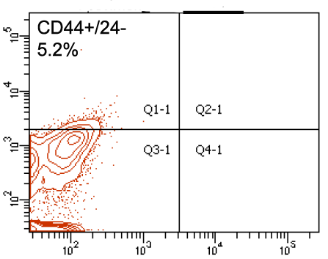

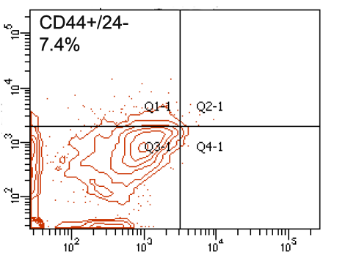

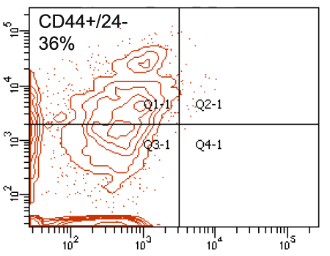


**MDA-MB-231**

**Mammospheres**

**Mammospheres + Pax**

**CD44 (PE-A)**

**CD24 (FITC-A)**


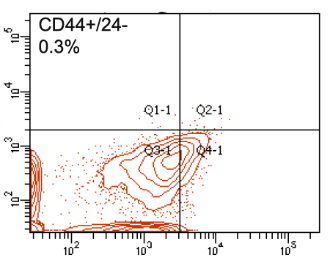

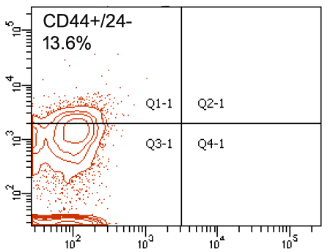


**CD24 (FITC-A)**

**CD44 (PE-A)**

**Normal**

**Tumor**

**CT-Tumor**


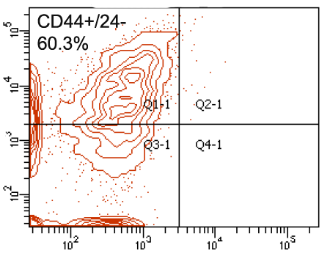


**Supplementary Figure 2 (S2): Immunophenotyping of CD44^+^/CD24^-^ populations from patient samples and triple negative cell line**. Immuno-staining and FACS analysis oftumor and chemo-treated tumors from patients undergoing MRM in comparison to the normal mammary tissue (panel A) and from MDA-MB-231 mammospheres with or without Pax treatment (panel B). CD44^+^/24^-^ subset of population increases with chemo-treatment (60.3%) as compared to a naive tumor (13.6%). Pax treatment also increases this subset in the stem cell compartment of MDA-MB-231 (36%) as compared to untreated mammospheres (7.4%) (n=30).

**B**


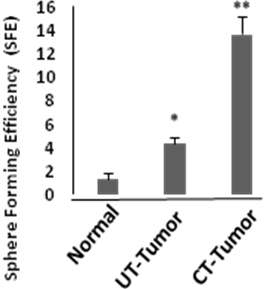


**Untreated Tumor**

**CT- Tumor**


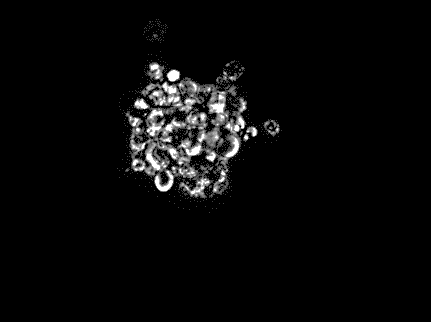

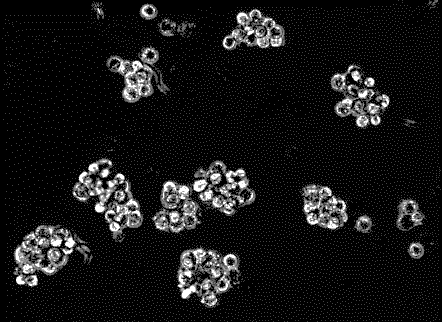


**Supplementary Figure 3 (S3): Sphere-forming efficiency in untreated versus chemo-treated (CT) tumor samples.** Chemotreatment of TNBC tumors increases the number of mammospheres from primary tumor sites. A subsequent increase of SFE (sphere forming efficiency) as compared to the naïve untreated tumor is also observed (n= 20). Scale bar 10 µM. Data is expressed as mean ± SEM of three independent experiments. Student’s t-test was used to calculate statistical significance. *p<0.05, **p<0.01 and ***p<0.001.


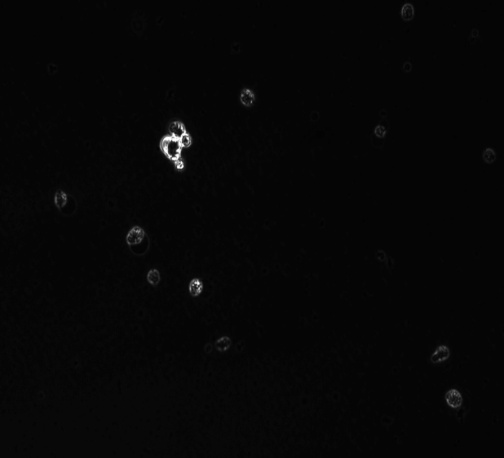

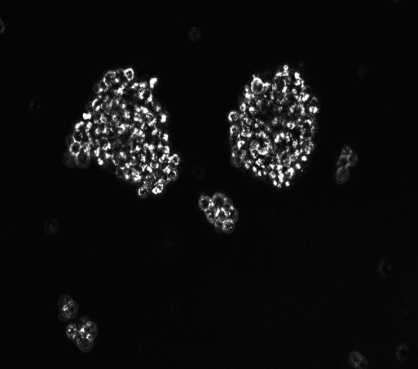

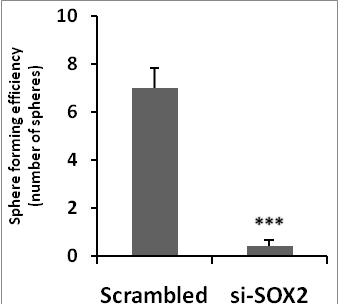


**Scrambled control**

**si-SOX2 treated**

**Supplementary Fig 4 (S4): Determining the self-renewal ability of SOX2-downregulated BrCSCs by sphere forming efficiency assays.** Mammosphere assay shows silencing SOX2 decreases the sphere forming efficiency in primary CT-TNBC tumors (n=10). Scale bar 10 µM. Data is expressed as mean±SEM of three independent experiments. Student’s t-test was used to calculate statistical significance. *p<0.05, **p<0.01 and ***p<0.001.


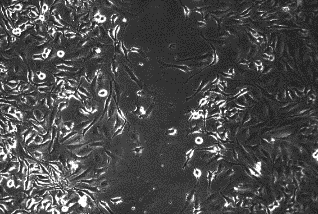


**24 hrs**


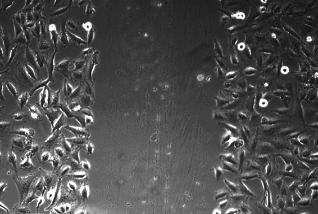

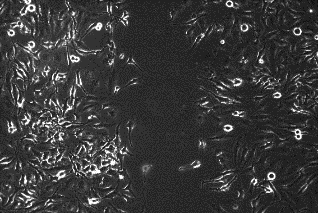

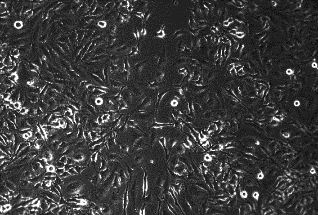


**48 hrs**


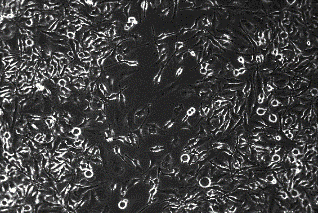

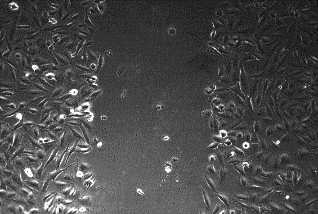

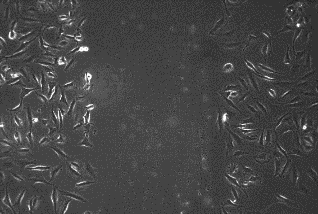

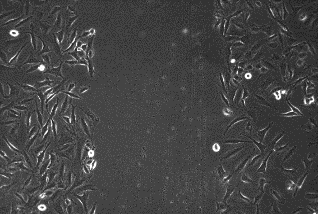

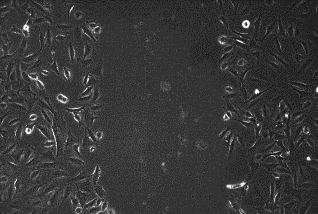


**0 hr**

**Supplementary Fig 5 (S5): Effect of SOX2 silencing in Pax removal sets of adherent MDA-MB-231 cultures.** Wound healing assays showing reduced migration of si-SOX2 Pax treated MDA-MB-231 cells compared to unsilenced cells after 24 hours (24 hours vertical panel). Inhibition of migration was sustainable even 24 hours after drug removal (2nM Pax) in SOX2 silenced MDA-MB-231 cells as compared to scrambled control (48 hours vertical panel).

**Scrambled**

**Scrambled**

**+Pax (24hrs)**

**-Pax (24 hrs)**

**Si-SOX2**

**+Pax (24hrs)**

**-Pax (24 hrs)**
